# End-Tidal Hypocapnia Under Anesthesia Predicts Postoperative Delirium

**DOI:** 10.3389/fneur.2018.00678

**Published:** 2018-08-17

**Authors:** W. Alan C. Mutch, Renée El-Gabalawy, Linda Girling, Kayla Kilborn, Eric Jacobsohn

**Affiliations:** ^1^Department of Anesthesia and Perioperative Medicine, Max Rady College of Medicine, University of Manitoba, Winnipeg, MB, Canada; ^2^Canada North Concussion Network (www.CNCN.ca), Winnipeg, MB, Canada; ^3^Department of Clinical Health Psychology, Max Rady College of Medicine, University of Manitoba, Winnipeg, MB, Canada

**Keywords:** postoperative delirium, anesthetic agent, carbon dioxide, hypocapnia, normocapnia, hypercapnia, cognition, postoperative complications

## Abstract

**Background:** Postoperative delirium (POD) might be associated with anesthetic management, but research has focused on choice or dosage of anesthetic drugs. We examined potential contributions of intraoperative ventilatory and hemodynamic management to POD.

**Methods:** This was a sub-study of the ENGAGES-Canada trial (NCT02692300) involving non-cardiac surgery patients enrolled in Winnipeg, Canada. Patients received preoperative psychiatric and cognitive assessments, and intraoperatively underwent high-fidelity data collection of blood pressure, end-tidal respiratory gases and anesthetic agent concentration. POD was assessed by peak and mean POD scores using the Confusion Assessment Method-Severity (CAM-S) tool. Bivariate and multiple linear regression models were constructed controlling for age, psychiatric illness, and cognitive dysfunction in the examination of deviations in intraoperative end-tidal carbon dioxide (areas over (AOC) and under the curve (AUC)) on POD severity scores.

**Results:** A total of 101 subjects [69 (6) years of age] were studied; 89 had comprehensive intraoperative hemodynamic and end-tidal gas measurements (data recorded at 1 Hz). The incidence of POD was 11.9% (12/101). Age, cognitive dysfunction, anxiety, depression, and intraoperative end-tidal CO_2_ (AUC) were significant correlates of POD severity. In the multiple regression model, cognitive dysfunction and AUC end-tidal CO_2_ (0.67 kPa below median intra-operative value) were the only independent significant predictors across both POD severity (mean and peak) scores. There was no association between cumulative anesthetic agent exposure and POD.

**Conclusions:** POD was associated with intraoperative ventilatory management, reflected by low end-tidal CO_2_ concentrations, but not with cumulative anesthetic drug exposure. These findings suggest that maintenance of intraoperative normocapnia might benefit patients at risk of POD.

## Introduction

Research has not explicitly focused on the effect of anesthetic management on postoperative delirium (POD) despite preliminary evidence that suggests this plays an important role (1–3). The healthcare costs and societal burden of POD are immense (2). Largely based on animal research, the problem is held, in part, to be a neurotoxic consequence of anesthetic agents. We examined intraoperative anesthetic course comprising ventilatory and hemodynamic management and their individual or combined potential for impact on POD as correlated to established preoperative risk factors.

Meta-analyses and comprehensive reviews have identified age, history of psychiatric illness, alcohol use, and cognitive dysfunction, as independent risk factors for POD (4, 5). An intraoperative stressor appears to act as a catalyst in the expression of POD. That said, POD also exists outside of surgical settings, as rates are high in intensive care units. We have recently hypothesized that the anesthetic agent might be a “strawman” (6) and suggest that intraoperative management of anesthesia is where the risk for POD potentially lies ‘(7, 8). Beyond safe administration of anesthetic agents to facilitate surgery, the conduct of anesthesia comprises intraoperative hemodynamic control and management of respiratory gas exchange while the patient is anesthetized. Very little attention has been paid to how end-tidal gas management impacts POD. It is well established that CO_2_ tension is a fundamental driver of cerebral blood flow [CBF; (9–12)]. Alterations in end-tidal CO_2_ can result in “intracranial steal” which is defined as a paradoxical decrease in regional CBF in response to a vasodilatory stimulus in the presence of vascular pathology. (11, 13, 14), In this context intracranial steal is a potential causal risk for POD. A large retrospective study indicated that hypocapnia, as determined by intraoperative measurement of end-tidal CO_2_, is associated with hospital length of stay and 30-day postoperative mortality (15). Their work suggests that perioperative ventilatory management as assessed by end-tidal CO_2_ can be predictive of patient outcome.

This hypothesis-generating study explores the relationships between intraoperative hemodynamic parameters, end-tidal CO_2_, and anesthetic agent on the severity of POD. We account for the effects of premorbid risk factors in order to understand possible independent and interactive effects of these intraoperative factors. The data examined are from a discontinued subset of patients enrolled in the ENGAGES-Canada study (NCT02692300). We hypothesize that when controlling for premorbid risk factors, deviations in end-tidal CO_2_ will independently predict POD severity.

## Methods

This study was approved by the Biomedical Research Ethics Board at the University of Manitoba. All patients recruited gave witnessed written informed consent. The study was conducted from February 22, 2016 to October 21, 2016.

### Selection criteria

The current study is a discontinued subset of the pragmatic ENGAGES-Canada study comprising both EEG-guided anesthetic management and current routine anesthetic management for non-cardiac surgery—a companion study to the ENGAGES study underway in the USA (16). The intervention was suggestive rather than prescriptive. This study was conducted at the University of Manitoba at two tertiary care hospitals. Inclusion criteria were: adults older than 60 years of age, competent to provide informed consent, undergoing elective non-cardiac surgery requiring a minimum stay of 2 days postoperatively. Exclusion criteria included patients undergoing neurosurgical procedures, presence of current preoperative delirium, patients unable to participate adequately in delirium screening including those who are blind, deaf, illiterate or not fluent in English, a history of intra-operative awareness, and patients undergoing a second pre-planned surgery within 5 days. Following recruitment of the first 101 subjects, an analysis was completed to examine separation of groups with respect to anesthetic depth (i.e., avoidance of burst suppression). There was no statistically significant separation of groups on the basis of EEG burst suppression [by BIS (bispectral index score—a processed EEG assessment)], and therefore recruitment was discontinued in this arm of the study (manuscript to describe these findings in preparation) and the ongoing Canadian trial arm focuses exclusively on cardiac surgery patients. This analysis is based on these first 101 subjects.

### Preoperative psychiatric and cognitive assessments

Following recruitment and informed consent in the Pre-Anesthetic Clinic (PAC), patients completed validated psychiatric and cognitive assessments. The Patient Health Questionnaire [PHQ-4; (17)] was used to assess severity of depressive and anxiety symptoms using a summary score. The Alcohol Use Disorders Identification Test [AUDIT-C; (18)] assessed history of alcohol misuse using a summary score. Trained research personnel administered the 5-min Short Blessed Test [SBT; (19)], which assessed global cognitive function including orientation, memory, concentration and attention. It is a valid measure to predict global cognitive impairment in older adults and is sensitive to mild, moderate, and severe cognitive impairments.

### Medical morbidity

Medical morbidity scoring was derived from chart review and assessed with the Charlson Co-morbidity Index [CCI; (16, 20) as a measure of preoperative vulnerability or diathesis (7) for POD.

### Medications

Perioperative medications included opioid dose in intravenous morphine equivalents, midazolam equivalents, ketamine, dimenhydrinate, and haloperidol. Dosage was assessed continuously for morphine equivalents given that all patients received this medication; all other medication was reported as “present” as only a proportion of patients are administered these medications.

### Intraoperative assessments

Management was randomized to EEG guided (BIS monitor) anesthesia or not. Where appropriate, regional anesthetic supplements were undertaken (nerve blocks or epidurals). All subjects received a general anesthetic—either sevoflurane or desflurane in air:O_2_. No subject was administered N_2_O. All subjects received intravenous supplements including propofol and midazolam for induction and muscle relaxants as required. All were tracheally intubated and mechanically ventilated and had arterial cannulation (unless otherwise noted) to record blood pressure continuously and ECG monitoring to record heart rate. Hemodynamics, end-tidal gas tensions (O_2_, CO_2_, and anesthetic gas) were recorded at 1 Hz using a data acquisition system (TrendFace Solo) and stored on a laptop computer. The data recorded included heart rate, systolic, diastolic and mean arterial pressure, respiratory rate, tidal volume, end-tidal O_2_, and CO_2_, end-tidal anesthetic gas, and BIS monitor output. The duration of time in the operating room was also recorded. All subjects were initially monitored in the postanesthesia recovery room (Postoperative Day 0) and then transferred either to surgical intensive care or inpatient surgical wards. Intraoperative data were examined and we report on median arterial pressure, median end-tidal CO_2_, end-tidal anesthetic concentration in vol% (desflurane concentration corrected to sevoflurane concentration). For blood pressure, the pressure below the 10th percentile and above the 90th percentile was determined for each subject as an index of hemodynamic instability. For end-tidal CO_2_, the median value for the entire course of the anesthetic period was determined for each subject as an index of ventilatory management. As an index of variability in intraoperative end-tidal CO_2_ control the duration above or below the patient's median CO_2_ by ±0.67 kPa for the conduct of the intraoperative course was determined for each subject (the area under the curve (AUC) or area over the curve (AOC), respectively, in kPa × sec). When available the recorded blood gas values for arterial CO_2_ were compared to the simultaneously obtained end-tidal CO_2_ measured during blood sample withdrawal. Total anesthetic concentration (vol%) × sec was calculated to measure anesthetic exposure with correction to age-adjusted MAC done *post-hoc*. These data were collated with Excel spreadsheets and transferred to SPSS for statistical analysis. In house macros were written in visual BASIC applications (VBA) to calculate the AUC and AOC outlined above and to determine summations of CO_2_ and blood pressure measures and cross-product output for AUC-Hypocapnia × AUC-blood pressure (BP), AUC-Hypocapnia × AOC-BP, AOC-Hypercapnia × AUC-BP, AOC-Hypercapnia × AOC-BP were calculated (kPa^2^ × sec^2^). The cross-product output permitted determination of simultaneous changes in CO_2_ and blood pressure.

### Assessment of postoperative delirium

A trained, blinded interviewer conducted the comprehensive CAM-S (21), a structured 10–15 min clinical interview, to assess severity of POD up to 5 days postoperatively, assessed once daily based on a time of convenience. The total long form severity score was based on a sum score that could range from 0 to 19 accounting for presence of either acute onset of change or symptom fluctuation in mental status, inattention, and either disorganized thinking or altered level of consciousness. We report peak postoperative severity score (i.e., peak delirium score) for each subject throughout their inpatient stay and the average severity score up to 5 postoperative days (i.e., mean delirium score) based on the CAM-S long form. For subjects who did not want to complete the extended interview, a short severity form was offered and these scores were subsequently weighted on the same metric as the long form. The mean score was calculated for only available data on each subject. For example, if there was a missing CAM-S assessment, or if subjects were discharged before day 5, the mean was evaluated based on the days available. We assessed POD on a continuum given that higher severity scores even in the absence of full POD are associated with higher risk of increased length of stay, increased healthcare costs, and postoperative admittance to nursing homes, functional, and cognitive decline, and death within 90 days postoperatively compared to lower scores (21); and that recent research suggests POD can be better understood on a continuum of severity (22). Further, use of a continuous outcome variable allows for enhancement of statistical power in more limited sample sizes (23). For descriptive purposes, the presence of full POD is also documented and indicated by the presence of either acute onset of change or symptom fluctuation in mental status, inattention, and either disorganized thinking or altered level of consciousness. When information was missing from the CAM-S in order to evaluate the presence of full POD, a chart review was conducted and information was corroborated with the CAM-ICU, which was conducted daily as standard practice.

### Analytic approach

Data were analyzed using SPSS (Version 24.0) software. Prevalence rate and mean (SD) are first reported for all variables among the entire sample, and bivariate correlations examined the relationship between both premorbid (including CCI) and intraoperative risk factors, and continuous severity POD measures (peak POD and mean POD scores). If significant results were indicated for intraoperative factors, we conducted a bivariate linear regression model followed by a multiple regression model controlling for premorbid age, psychiatric illness, and cognitive dysfunction. Multiple linear regressions examined potential interactive relationships of end-tidal CO_2_ and all other primary variables, and main effects were included in both an unadjusted and adjusted model for the aforementioned covariates. Correlations between post-induction arterial and end-tidal CO_2_ were undertaken by linear regression and Bland-Altman analysis.

## Results

**Figure 1:**
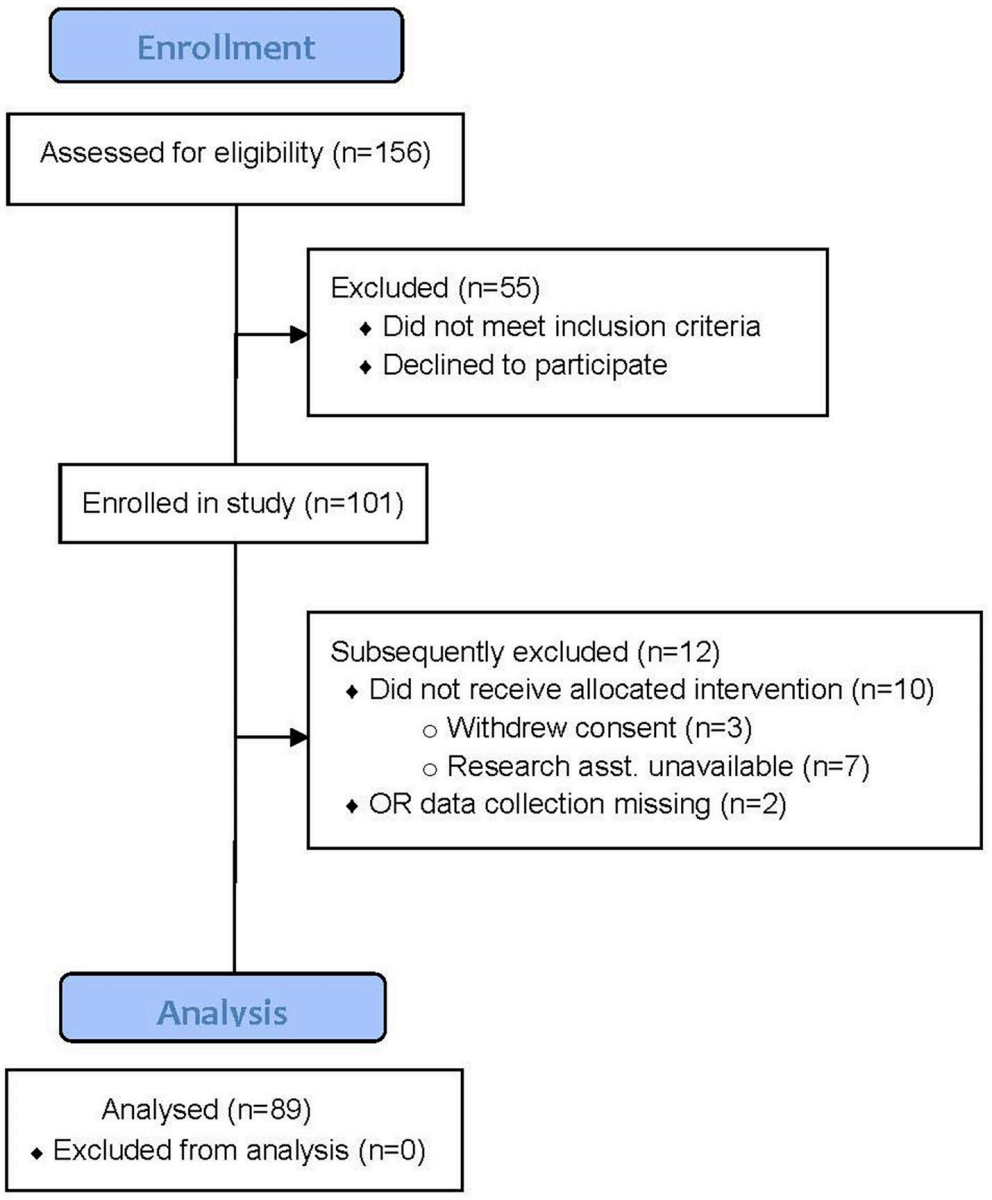
CONSORT Diagram for the study. In total 101 subjects (intention to treat) were studied of which 89 had intraoperative hemodynamic and end-tidal gas data recorded. There were 84 subjects with full hemodynamic and end-tidal gas data; in 5 only non-invasive BP was measured. These 5 subjects do not have data available for cross-product calculations discussed in the text.

The CONSORT diagram describing subject recruitment and allocation is shown in Figure 1. Subjects represent a convenience sample drawn from a subset of the ENGAGES-Canada study. There were very few missing data on all primary variables (<5%), therefore these data were treated as a pairwise deletion. Full intraoperative data were only available for 89 subjects, and therefore this subsample was evaluated in analyses including these factors. In five subjects only non-invasive blood pressure measurements were recorded, so only end-tidal respiratory gases and anesthetic concentration data are reported.

A total of 12 subjects met full criteria for incident POD (11.9%; 12/101). Those with full POD had significantly greater mean peak 8.5 (2.5); *t*_(94)_ = −7.7, *p* < 0.001 and mean 5.4 (2.1); *t*_(94)_ = −5.4, *p* < 0.001 POD scores than those without POD. The POD subjects spent an average of 5.4 (1.2) days in hospital.

Subject characteristics and results of bivariate correlations are shown in Table 1 for POD severity among the entire sample. Subjects were 68.7 (6.4) years old, and primarily male (64.4%). The majority (78.4%) were tracheally extubated in the operating room. There were no significant differences in tracheal extubation location (operating room or postanesthesia recovery room, or intensive care unit) on POD severity.

**Table 1:** Premorbid, surgical, and delirium characteristics of the sample and their association to postoperative delirium severity.

		**Correlation coefficient**	
		**POD severity**	
	**Total sample (*n* = 101)**	**Peak score**	**Mean score**
**SOCIODEMOGRAPHICS**			
Age, years (range = 60-86)	68.7 (6.4)	0.06	0.21^*^
Male Sex, *n* (%)	65 (64.4%)	−0.13	−0.12
**PREOPERATIVE COGNITIVE FUNCTIONING**			
Short Blessed Test, score (range = 0–21)	2.0 (3.1)	0.29^**^	0.15
**PREOPERATIVE PSYCHIATRIC CHARACTERISTICS**			
PHQ Total, score (range = 0–10)	2.4 (2.7)	0.27^**^	0.32^**^
Alcohol Use, score (range = 0–9)	2.1 (2.2)	−0.01	0.03
**PREOPERATIVE PATIENT MORBIDITY**			
Charlson Comorbidity Index (range = 0–9)	6.3 (2.5)	−0.04	−0.01
**SURGICAL CHARACTERISTICS**			
Duration of Anesthesia (min)	208.8 (101.9)	−0.12	−0.10
Mean Arterial Pressure, mm Hg	78.4 (7.9)	−0.01	0.05
End–Tidal CO_2_, kPa	4.50 (0.4)	0.05	−0.01
Anesthetic agent (vol%)	1.2 (0.3)	−0.13	−0.19
Anesthetic dose × time (vol% sec)	14994 (8037)	−0.13	−0.17
AUC Blood Pressure (kPa sec)	5080 (2868)	−0.002	0.02
AOC Blood Pressure (kPa sec)	9124 (7193)	−0.04	−0.10
AUC CO_2_-0.67kPa	350 (1196)	0.25^*^	0.26^*^
AOC CO_2_-0.67kPa	2453 (3300)	−0.04	0.01
***Medications***			
Morphine equivalents (range = 2–1509 mg)	68.8 (156.6)	0.14	0.17
Benzodiazepines, presence *n* (%)	32 (33.7%)	0.11	0.03
Ketamine, presence n (%)	20 (21.1%)	0.08	−0.01
Haloperidol, presence n (%)	4 (4.2%)	0.15	0.14
Dimenhydrinate, presence n (%)	25 (26.3%)	0.17	0.10
**DELIRIUM**			
***Preoperative***			
History of Previous Delirium, *n* (%)	14 (13.9%)	0.05	0.17
***Postoperative***			
CAM-S Peak Score	4.2 (2.6)	–	0.89^***^
CAM-S Mean Score	2.5 (1.8)	0.89^***^	–
Days Delirious	0.2 (0.7)	0.59^***^	0.67^***^

* p < 0.05,

** p < 0.01,

*** *p < 0.001*.

*Data are presented as mean (SD) unless otherwise specified and Pearson's correlation coefficient is derived. A point-bi-serial correlation coefficient is reported for dichotomous variables. Perioperative characteristics were evaluated for n = 89 participants. CAM-S, Confusion Assessment Method-Severity Score; AUC, area under the curve; AOC, area over the curve; POD, postoperative delirium. The bold text highlights the statistically different results*.

The operative intervention for the subjects enrolled is shown in Supplemental File 1. Of the assessed premorbid and intraoperative factors, age, cognitive dysfunction, anxiety, and depressive symptoms, and intraoperative CO_2_ were significant correlates of POD severity. Alcohol use was not significant, and therefore not included in multiple regression models. All other premorbid and surgical factors including medical morbidity as assessed by the CCI, and medication (drug dosage equivalence; shown in Supplemental File 2) were not significant, and therefore not included in subsequent multiple regression models. There was no difference between patients with POD and those without for benzodiazepine administration, either midazolam equivalents or longer acting agents such as diazepam or lorazepam.

**Figure 2:**
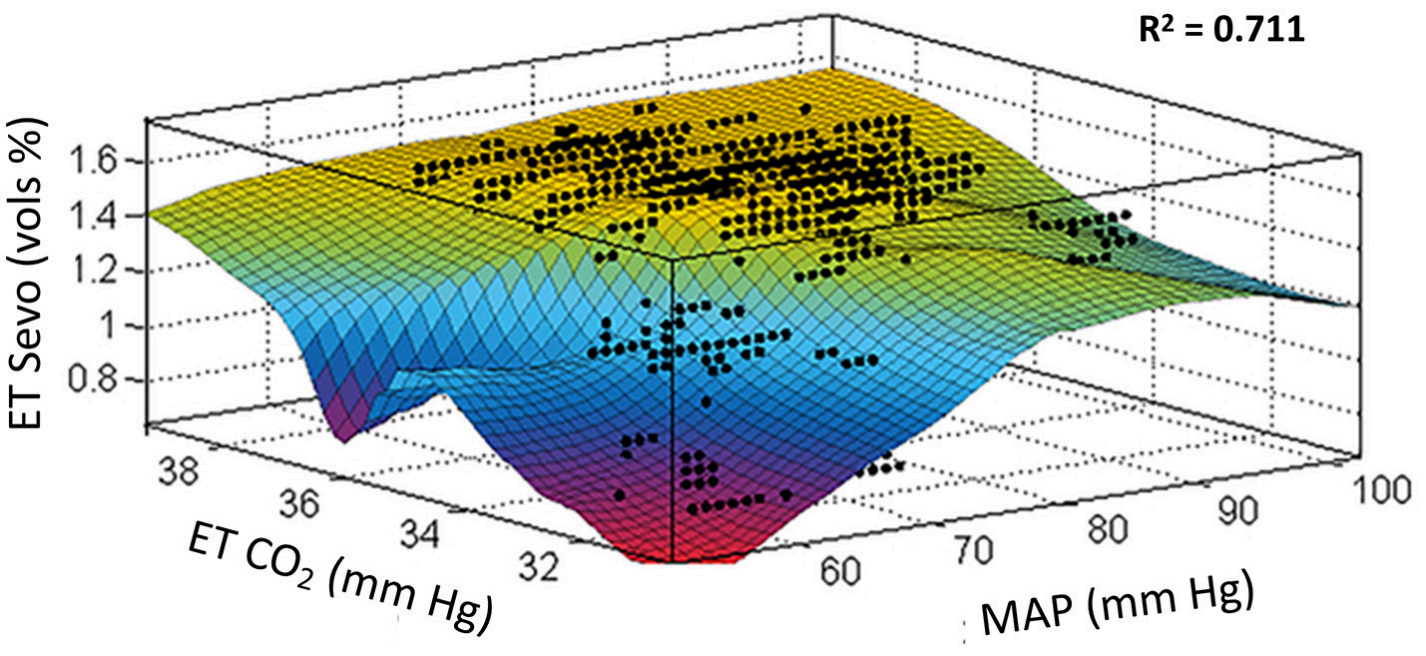
Locally weighted smoothing linear regression. Goodness of fit: SSE: 137, R^2^: 0.711, Adjusted R^2^: 0.710, RMSE: 0.112. These data were fit from one subject with high-fidelity recording for the variables shown totaling 10,997 data points (data collected at 1 Hz for a duration of slightly more than 3 h of intraoperative time). This study indicates that the area of “roll-off” where the output changes to blue then red places the patient at risk of developing POD. The period of hypocapnia (instability re baseline normocapnia) especially in the presence of hypotension is an intraoperative marker for POD. x, mean arterial pressure; y, end-tidal CO_2_; z, sevoflurane concentration.

High-fidelity data collection is shown for a representative subject in Figure 2. This 3-dimensional plot shows the interaction between MAP, end-tidal CO_2_, and end-tidal anesthetic concentration during the conduct of anesthesia.

**Figure 3:**
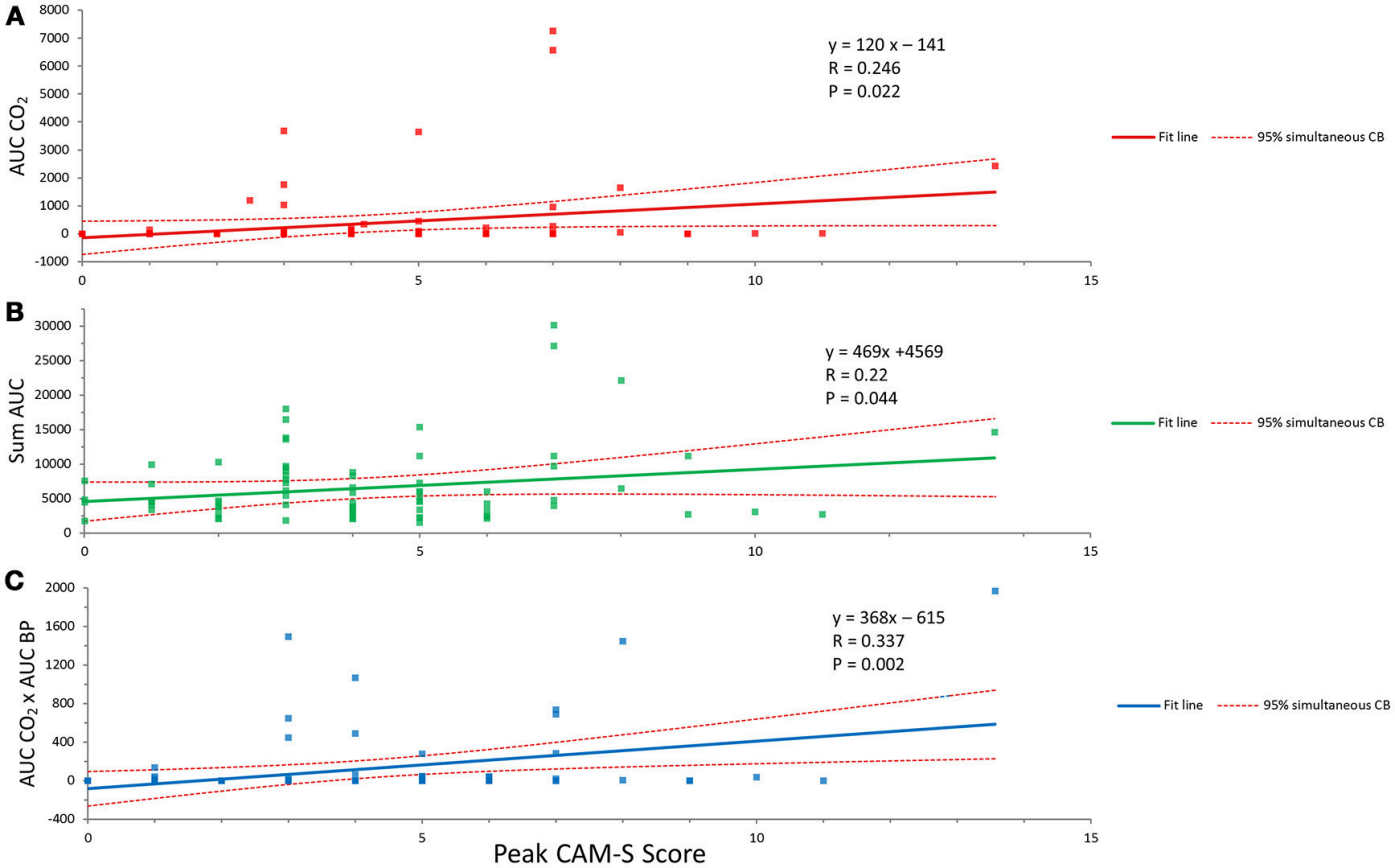
Relationships between hypocapnia and POD. **(A)** The correlation between hypocapnia (AUC CO_2_ less than 0.67 kPa from individual median value and peak CAM-S score as an index of POD). **(B)** Summation of hypocapnia and hypotension and the peak CAM-S score. **(C)** Simultaneous hypocapnia and hypotension and peak CAM-S score. See text for further details.

The collective intraoperative data for the various relationships with AUC CO_2_ for 0.67 kPa below the individual median value are shown in Figures 3A–C. A significant correlation is seen for hypocapnia of this magnitude or greater and peak CAM-S scores; correlations are also seen for the sums of AUC CO_2_ and AUC BP and the interaction between AUC CO_2_ and AUC BP. In the latter circumstance hypocapnia and hypotension were occurring simultaneously. There were no similar statistically significant correlations for AOC CO_2_ for 0.67 kPa above the individual median value collectively or for summations or interactions between hypercapnia and hypertension.

**Figure 4:**
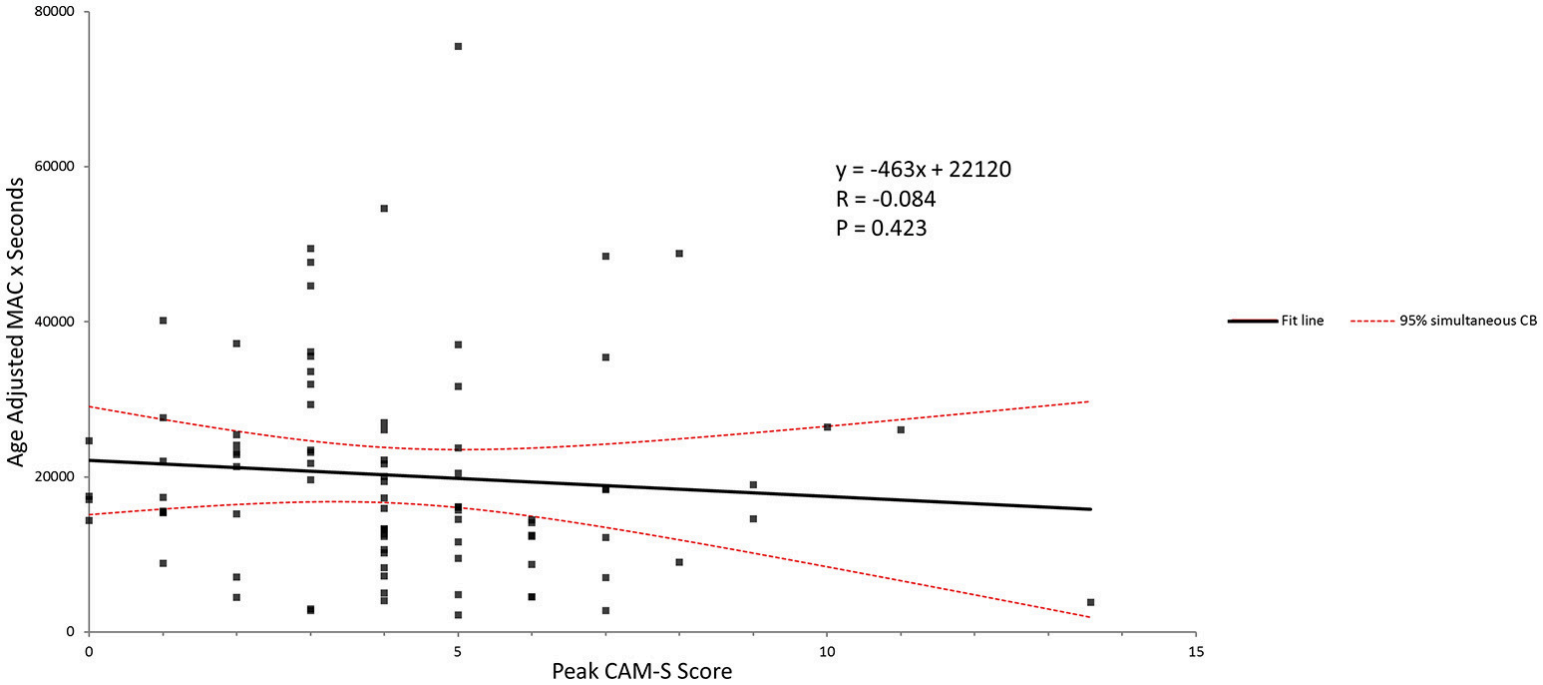
Age-adjusted MAC and peak CAM-S score. There was no correlation seen between anesthetic dose-duration and the POD score.

Figure 4 shows the age adjusted MAC × duration of anesthesia versus peak CAM-S scores for the complete patient group. No correlation was seen here.

The relationship between post-induction arterial and end-tidal CO_2_ is shown in Supplemental File 3. The Bland-Altman plot (A) shows a bias of 1.12 kPa with the greater value seen with arterial measure. The correlation (B) between the two measures was R^2^ = 0.34.

The dynamic nature of alterations in ΔCO_2_ are commented on in Supplemental File 4 and how this instability of intraoperative CO_2_ over time can contribute to intracranial steal.

Table 2 shows results of the various regression analyses. The assumptions of linear regression were tested and were met; thus, statistical models appear valid. Intraoperative indices of hemodynamic stability for AUC and AOC below the 10th percentile and above the 90th percentile for each POD condition, deviation from the index of normocapnia (median CO_2_); AUC for median CO_2_ less by 0.67 kPa and AOC median CO_2_ greater by 0.67 kPa were used as the intraoperative predictors in analyses given consistent findings across both POD severity measures in bivariate correlations in Table 1. Results revealed that in the adjusted multiple regression model, cognitive dysfunction and AUC CO_2_ were the only independent significant predictors across both severity POD scores (Table 2). When examining AOC CO_2_ in the models, only cognitive dysfunction was a significant independent predictor across both severity models. Randomization groups based on the larger ENGAGES-Canada trial were also included in the most stringent model as a validity check and did not impact any of the findings (not reported). Results of interaction analyses revealed that AUC BP × AUC CO_2_ was significantly associated with peak POD severity (*Beta* = 0.337, *p* = 0.013) and mean POD severity (*Beta* = 0.329, *p* = 0.015). Results of interaction terms remained significant when included in the multiple regression model adjusting for age, psychiatric status, and cognitive dysfunction for both peak POD severity (*Beta* = 0.361, *p* = 0.005) and mean POD severity (*Beta* = 0.334, *p* = 0.007). No other interaction terms were significant.

**Table 2:** Bivariate and multiple linear regressions examining the relationship between significant predictors and delirium severity.

	**POD peak score**				**POD mean score**			
	**Model 1**		**Model 2**		**Model 1**		**Model 2**	
	**Beta**	***P-Value***	**Beta**	***P-Value***	**Beta**	***P-Value***	**Beta**	***P-Value***
**AUC CO**_2_ 0.67 kPa
Age	0.055	0.593	0.067	0.521	0.213*	0.038	0.223*	0.035
Short Blessed Test Score	0.286**	0.005	0.338**	0.001	0.149	0.150	0.220*	0.034
PHQ total score	0.273**	0.008	0.145	0.159	0.323**	0.002	0.185	0.075
AUC CO_2_	0.246*	0.020	0.220*	0.033	0.261*	0.013	0.205*	0.048
**AOC CO**_2_ 0.67 kPa
Age	0.055	0.593	0.103	0.334	0.213*	0.038	0.250*	0.020
Short Blessed Test Score	0.286**	0.005	0.334**	0.002	0.149	0.150	0.215*	0.044
PHQ total score	0.273**	0.008	0.184	0.080	0.323**	0.002	0.219*	0.038
AOC CO_2_	−0.042	0.695	−0.052	0.613	0.007	0.951	−0.034	0.742

* p < 0.05,

** *p < 0.01*.

*Standardized betas are reported. Model 1, unadjusted bivariate regressions examining each independent predictor. Model 2, multivariable model including all listed predictors in single model. All covariates entered in models were assessed continuously. CO_2_, carbon dioxide; AUC, area under the curve; AOC, area over the curve; PHQ, patient health questionnaire; POD, postoperative delirium. The bold text highlights the statistically different results*.

## Discussion

This represents the first study to identify intraoperative end-tidal hypocapnia (i.e., AUC CO_2_) as an independent risk factor of POD severity. There was also a significant interaction between hypocapnia and hypotension and more so when hypocapnia and hypotension occurred simultaneously. In contrast, volatile anesthetic exposure duration was not significant. Independent preoperative predictors of POD were advanced age, cognitive dysfunction and psychiatric status, in line with prior research. We identified an incident rate of POD for this adult population of 11.9%; severity scores were significantly greater for these individuals.

It has been known that hypocapnic CO_2_ values can correlate to morbidity or mortality (24, 25). An older literature, some dating back over 50 years, has indicated that pronounced intraoperative hypocapnia (more severe than examined here) can impair some indices of cognitive performance postoperatively (26–28). A large retrospective study by Dony et al. (15) indicates that intraoperative hypocapnia is associated with greater length of stay and greater 30 day mortality after non-cardiac surgery in adults. Their study in 5317 patients indicated that nearly 2/3 patients were hypocapnic by their criteria (end-tidal CO_2_ < 4.7 kPa) at a mean end-tidal CO_2_ the same as described here (4.4 kPa). Our study extends their findings to indicate that similar levels of hypocapnia can result in perioperative cognitive dysfunction as well. Importantly we did not find significant confounders to this assessment. There were no significant differences between subjects with POD and pre-morbid medical conditions or medications, intraoperative use of benzodiazepines or ketamine, nature of the surgical intervention or morphine equivalents of opioids administered postoperatively.

Change in CO_2_ is an important driver of alterations in cerebral blood flow (CBF) with normal physiology. Increasingly, it is clear that changes in CO_2_ (ΔCO_2_) can result in intracranial steal with brain-at-risk (11, 29). Further discussion of this issue can be found in Supplemental File 4. This situation arises when CBF transitions from hypocapnia to normocapnia and from normocapnia to hypercapnia. Both of these situations were documented in our subjects. The latter situation was particularly evident at the end of the surgical procedures when spontaneous ventilation was being re-established. Intraoperative transition from hypocapnia to normocapnia with intraoperative steal could be intensified in the presence of volatile anesthetic with its known effect to decrease cerebrovascular tone in normal vessels. Less effect of volatile anesthetic on cerebrovascular tone occurs with hypercapnia, and in this study hypercapnia at the end of the procedure was associated with precipitous decreases in anesthetic concentration, limiting the potential interaction between hypercapnia and the anesthetic agent. We have limited our examination of the effects of CO_2_ on a ΔCO_2_ of 0.67 kPa from the median value. We suggest that this degree of end-tidal CO_2_ variability is a viable target goal for intervention and in evidence with a subpopulation of the cases examined.

Greater cognitive dysfunction was the most robust preoperative marker correlated with POD, which independently predicted POD severity with intraoperative hypocapnia. Dementias and various other neurological conditions are associated with changes in cerebrovascular responsiveness (30–32).

The finding that hypocapnia was correlated with POD is further strengthened by the summative effects with hypotension and the greater association when the two effects occurred simultaneously (see Figures 3A–C).

Preoperative screening for POD could be greatly helpful to permit optimal anesthetic management in at risk patients. The Short Blessed Test was a robust indicator of risk of POD in this study. It is an easy and short screening test and can be completed in ~5 min. Not examined in any detail in the current study is the linkage between preoperative risk and modification of POD risk with anesthetic management. Patients may be seen to stratify into three potential groupings—high risk for POD based on preoperative psychiatric and cognitive status, those at moderate risk and those at low risk. Intraoperative management might have limited impact on those at high risk—but poor intraoperative management might affect severity of POD. The low risk group might be inherently unaffected by intraoperative management. The greatest benefit of optimized anesthetic management might be in patients at moderate risk of POD. These concepts fit into a stress-diathesis (vulnerability) model whereby the stress (operative intervention) is modified and declared, in part, by the diathesis (7).

The findings of this study should be considered in light of its limitations. First, this analysis was based on a secondary aim of ENGAGES-Canada and therefore a priori guidelines specific to this study were not established, nor was an a priori power analysis conducted. Second, although validated, preoperative psychiatric measures were based on self-report, which may result in biased estimates. Third, the Short Blessed Test does not comprehensively assess specific facets of cognitive functioning. However, as a cognitive assessment tool, it can feasibly be implemented as a screening measure. Fourth, only breath-by-breath end-tidal CO_2_ was comprehensively assessed, which might underestimate blood concentrations of CO_2_ or ΔCO_2_ in comparison to arterial CO_2_ blood gas determinations, but end-tidal CO_2_ offers the considerable advantage of continuous measurement (see Supplemental File 3 for additional discussion). Where simultaneous assessments of arterial and end-tidal CO_2_ were compared our results indicate a good correlation with a stable bias over the range of CO_2_ values examined. The important corroborating study by Dony et al. (15) indicating worse outcome with hypocapnic end-tidal CO_2_ is noted. Finally, POD and POD severity were only assessed once daily at a time of convenience, which might not accurately represent the full spectrum of POD as it presents with a fluctuating course. Although both POD peak and mean were included in this study, future research should aim to examine POD more rigorously, with several assessments throughout postoperative days.

In conclusion, we found in older patients undergoing non-cardiac surgery that duration and severity of intraoperative hypocapnia was independently associated with severity of POD. Maintenance of normocapnia is likely to be achievable during general anesthesia. As such, intraoperative hypocapnia is potentially a modifiable factor for POD. Before recommending specific changes in anesthetic practice, it would be important to test in a rigourous clinical trial enrolling older adults at risk for POD whether tight intraoperative maintenance of normocapnia is (i) feasible, (ii) decreases incidence or severity of POD, and (iii) does not lead to unforeseen adverse events or outcomes. These novel findings suggest that intraoperative stability of arterial CO_2_ is an important factor to examine in perioperative research focusing on POD and other poor surgical outcomes.

## Author contributions

WACM, RE-G, EJ: conception and design, analysis and interpretation of data, drafting and revising article, final approval, accountable for all aspects of the work. LG and KK: acquisition of data, analysis and interpretation of data, revising article, final approval, accountable for all aspects of the work.

### Conflict of interest statement

The authors declare that the research was conducted in the absence of any commercial or financial relationships that could be construed as a potential conflict of interest.
