# Supplementary material for: End-Tidal Hypocapnia Under Anesthesia Predicts Postoperative Delirium
Source: Front Neurol. 2018 Aug 17;9:678. doi: 10.3389/fneur.2018.00678 (PMC6108130; doi:10.3389/fneur.2018.00678)
Supplement: Supplementary file 1 [file Data_Sheet_1.PDF]

Supplemental File 1 – Intention to Treat

Study	Surgical Procedure
1	Cholecystectomy; Gastrojejunostomy; Hepatojejunostomy
2	Esophagectomy
3	Open AAA Repair
4	Laparotomy and resection of GIST tumor of stomach
5	Aortobifemoral Bypass; Right femoral artery endarterectomy
6	Sigmoid Resection
7	Transduodenal Exploration of Common Bile Duct; Intraop Choledoscopy
8	Anterior Perineal Resection
9	Excision Retroperitoneal Sarcoma; Incisional Hernia Repair
10	N/A
11	Laparotomy ; Right Hemicolectomy
12	Radical Retropubic Prostatectomy with Bilateral Lymphadenectomy
13	Right thoracotomy; Right Pneumonectomy; Intrapericardial Dissection
14	Right hemicolectomy
15	Exploratory Laparotomy; Cholecystectomy; Lymph Node Biopsy
16	Laparotomy; Removal of Retroperitoneal Mass
17	Small Bowel Resection (Ileostomy) and Primary Anastomosis; Liver Lobectomy
18	N/A
19	Laparotomy; Transduodenal Resection of the Ampulla of Vater and Sphincteroplasty
20	Open AAA Repair
21	Splenectomy
22	Distal Pancreatectomy and Splenectomy
23	Distal Pancreatectomy, Splenectomy and Liver biopsies
24	Radical Prostatectomy with Bilateral Lymphadenectomy
25	Diagnostic Laparoscopy and Open Cholecystectomy
26	Left thoracotomy for left lower lobe lobectomy of lung
27	Juxtarenal Aortic Aneurysm Repair, Suprarenal Clamp
28	Abdomino-Perineal Rectal Resection
29	Open AAA Repair
30	Pancreaticojejunostomy
31	Left Thoracotomy for left upper lobectomy of lung
32	Left Thoracotomy; LLL Wedge Resection of Lung
33	Left Thoracotomy for Decortication of Left Fibrothorax; Intercostal Nerve Blocks
34	Small Bowel Resection
35	Revision Spinal Fusion
36	Hemicolectomy
37	Abdominal-Perineal resection
38	N/A
39	N/A

**40**	Whipple Procedure
**41**	Whipple Procedure
**42**	Lower Anterior Resection
**43**	Laparotomy for Right Trisegmentectomy of Liver
**44**	Right Thoracotomy for Right Upper Lobe Wedge Resection
**45**	Right hemicolectomy and mesenteric lymph node biopsy
**46**	Right hemicolectomy and Retroperitoneal Liposarcoma Resection
**47**	Laparotomy; Resection of GIST tumor of the stomach
**48**	Repair of Aorto-iliac aneurysm ; Left renal artery endarterectomy and bypass
**49**	Cystectomy; Ileal Conduit; Pelvic Exenteration and Low Anterior Resection
**50**	Total Gastrectomy
**51**	Laparotomy; Cholecystectomy; Liver Resection and RFA
**52**	Open Cholecystectomy
**53**	Low Anterior Perineal Resection and Ostomy
**54**	Posterior Cervical Decompression with Fusion and Laminectomy C4-C6
**55**	Laparotomy; Recto-sigmoid resection and diverting ostomy
**56**	Abdominal Perineal Rectal Resection
**57**	Abdominoperineal Resection
**58**	L2-L3 Laminectomy
**59**	Open AAA Repair
**60**	Laparotomy; Distal Pancreatectomy
**61**	TAH, BSO
**62**	Radical prostatectomy
**63**	Restorative proctocolectomy
**64**	Radical cystectomy
**65**	Total abdominal hysterectomy
**66**	TAH, BSO, PLND
**67**	AAA repair
**68**	Ileostomy reversal
**69**	Interval debulking, TAH, BSO
**70**	AAA repair
**71**	AAA repair
**72**	AAA repair
**73**	Exploratory laparatomy
**74**	Left hemicolectomy
**75**	Abdominal perineal resection
**76**	N/A
**77**	Radical prostatectomy with bilateral lymph node dissection
**78**	Radical prostatectomy
**79**	Radical prostatectomy
**80**	AAA repair
**81**	Radical prostatectomy with bilateral lymph node dissection

**82**	N/A
**83**	Radical prostatectomy with bilateral lymph node dissection
**84**	Radical prostatectomy with bilateral lymph node dissection
**85**	Low anterior resection, illeostomy
**86**	Pelvic exenteration with urinary ileal conduit, closure of transverse loop colostomy
**87**	Low anterior resection
**88**	Aorto biiliac aneursym repair
**89**	Revision parathyroidectomy
**90**	Allograft nephrectomy
**91**	Radical prostatectomy with bilateral lymph node dissection
**92**	Right nephroureterectomy with ileal conduit, cystectomy, prostatectomy
**93**	Exploratory laparotomy, lysis of adhesions, diverting transverse loop colostomy
**94**	Open right hemicolectomy
**95**	TAH, BSO, bilateral pelvic lymph nodes dissection
**96**	Abdominal perineal resection
**97**	Radical cystectomy,pelvic exenteration, ileal conduit
**98**	Low anterior resection with ileostomy
**99**	TAH, BSO, bilateral pelvic lymph nodes dissection
**100**	Open incisional hernia repair, reversal of ileostomy
**101**	Radical cystectomy

Non POD No post-operative delirium

POD post-operative delirium
