# Supplementary material for: End-Tidal Hypocapnia Under Anesthesia Predicts Postoperative Delirium
Source: Front Neurol. 2018 Aug 17;9:678. doi: 10.3389/fneur.2018.00678 (PMC6108130; doi:10.3389/fneur.2018.00678)
Supplement: Supplementary file 2 [file Data_Sheet_2.PDF]

# Supplemental File 2 - Peri-operative Medications

| | Cohort | POD |
|---|---|---|
| **Morphine** | | |
| mean | 69 | 72 |
| SD | 157 | 55 |
| **Midazolam** | | |
| mean | 1 | 1 |
| SD | 3 | 1 |
| **Ketamine** | | |
| mean | 7 | 6 |
| SD | 21 | 12 |
| **Haloperidol** | | |
| mean | 0 | 0 |
| SD | 0 | 0 |
| **Dimenhydrinate** | | |
| mean | 24 | 54 |
| SD | 56 | 96 |

Drug dosages in milligrams (mg)

Morphine = intravenous morphine equivalents
Midazolam = intravenous midazolam equivalents
Ketamine = intravenous equivalents

There were no statistical differences between groups – all cases
See Table 1 in manuscript for regression analyses
