# Supplementary material for: End-Tidal Hypocapnia Under Anesthesia Predicts Postoperative Delirium
Source: Front Neurol. 2018 Aug 17;9:678. doi: 10.3389/fneur.2018.00678 (PMC6108130; doi:10.3389/fneur.2018.00678)
Supplement: Supplementary file 3 [file Data_Sheet_3.pdf]

Supplemental File 3:

The relationship between $ETCO_2$ and $PaCO_2$

The conclusions arrived at in this paper indicate that instability of intra-operative $ETCO_2$ are associated with post-operative delirium (POD) predicated on alterations in cerebral blood flow (CBF). The real driver of alterations in CBF is the arterial CO_2 ($PaCO_2$) as measured by arterial blood gas (ABG). Thus a correlation between the $ETCO_2$ and $PaCO_2$ would further endorse our results based on the observations of increased incidence of POD for AUC for hypocapnia and the cross-product of AUC for hypocapnia and hypotension. Below are a series of paired observations of $ETCO_2$ and $PaCO_2$ obtained from various of the patients studied.

In 55 patients we have accurate time stamped ABGs obtained post-induction compared to matched measures of $ETCO_2$ as assessed by the data retrieval of high fidelity intra-operative recording of the end-tidal gases. As these were elective surgical patients these data were obtained when the patients were hemodynamically stable post-induction. The correlation between the two measures is shown in Figure 1.

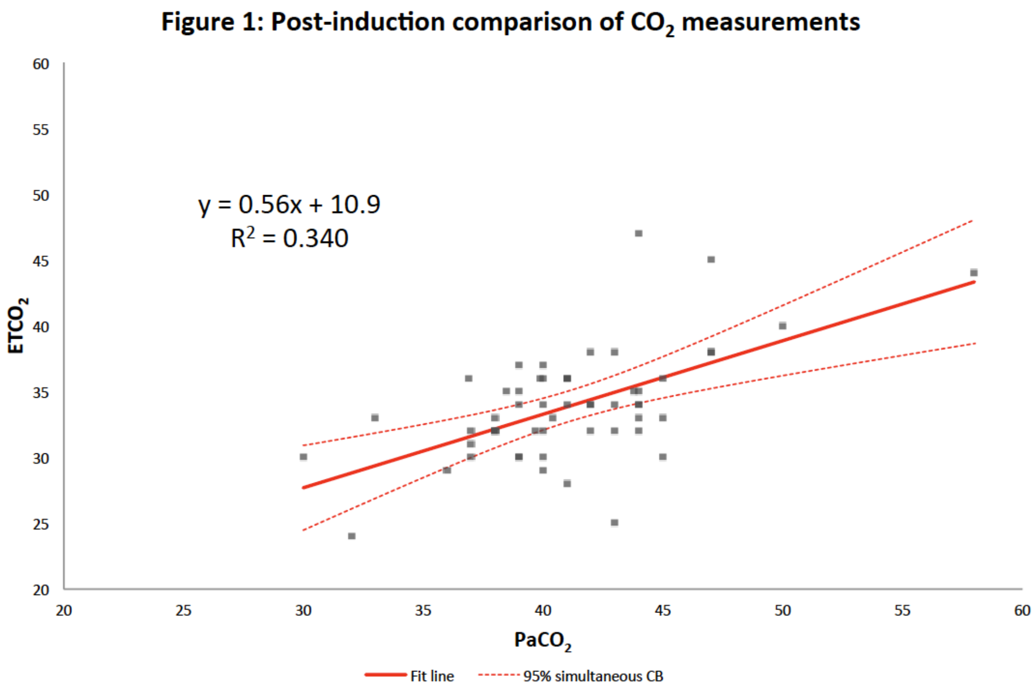

These data indicate a good correlation between $ETCO_2$ and $PaCO_2$ when the patients were hemodynamically stable and in presumed steady state at the beginning of the operative period. The correlation co-efficient (r) for these data is 0.63 with $p<0.0001$. The y-intercept indicates a significant offset or bias between the two measures with the $PaCO_2$ being higher.

A Bland-Altman plot of these same data is shown in Figure 2. The bias is 8.45 in this plot with the $PaCO_2$ being higher for any given measurement of $ETCO_2$. The mean/median $ETCO_2$ for the entire patient cohort was 33 mm Hg. This indicates that the corresponding $PaCO_2$ on average would be 41.5 mm Hg – well within the value for normocapnia for the total study population. This bias is a relatively high value and likely due to the fact that these are older patients mean age 69 years many with multiple co-morbidities (see Supplemental File 2) that can affect lung perfusion with changes in deadspace ventilation. These data also indicate that there is no obvious additional bias at either extreme – with hypocapnic and hypercapnic means not deviating significantly from the normocapnic values mid-range.

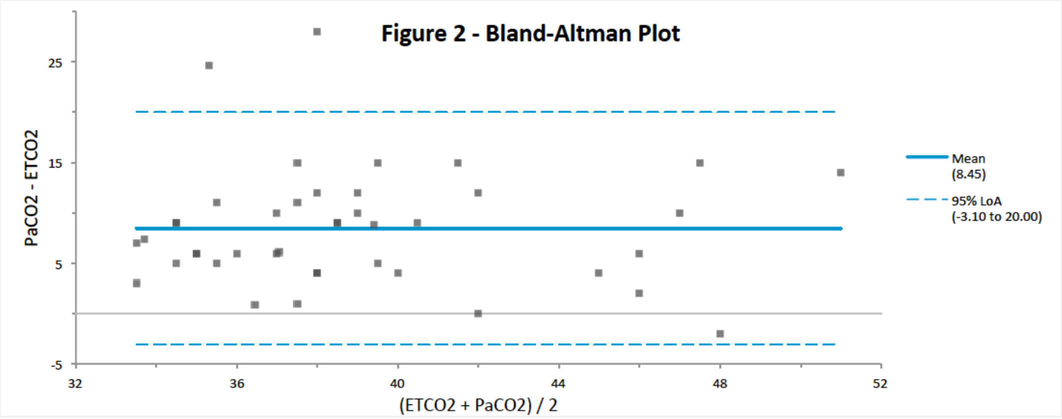

While the above 2 graphs indicate that $ETCO_2$ can be used as a surrogate for $PaCO_2$ in stable conditions our hypothesis is that it is the instability of intra-operative CO_2 which is contributory to the development of POD in the patients studied. Therefore we examined a series of our patients whom had paired measures of $ETCO_2$ and $PaCO_2$ at baseline as noted above and with a second paired measure intra-operatively. We obtained these data in 22 patients with 44 data points. The correlation is shown in Figure 3.

A correlation still exists but is less robust than seen in Figure 1. The correlation co-efficient is now 0.37 and the p-value for these data is 0.019. Also evident is that the y-intercept is now higher at 19 mm Hg. Thus in the context of varying blood pressure the correlation between $ETCO_2$ and $PaCO_2$ is not as robust and $ETCO_2$ declares as a lower value for a given $PaCO_2$. This finding is in agreement with our

Figure 3 - Paired CO_2 comparisons for CO_2 measurement technique

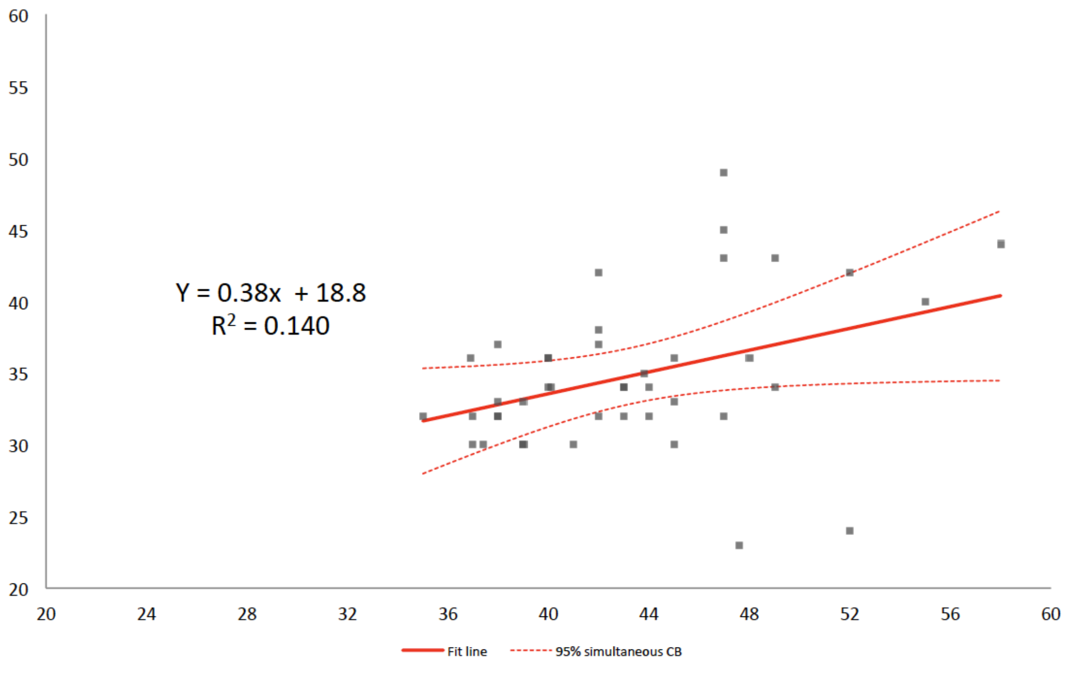

thesis that it is low $ETCO_2$ that we have identified as a marker for POD. It remains possible as indicated here that the $PaCO_2$ as a biomarker for POD may be higher. However, our thesis advances the concept that it is the instability of CO_2 that is the potential driver of POD with the changes in CO_2 increasing the risk of intra-cranial steal of CBF in susceptible areas. There is evidence here that $ETCO_2$ monitoring is an accurate trending monitor of CO_2 changes in the presence of alterations in blood pressure.

Below (Figure 4 A and B) is an examination of the interaction between changes in CO_2 and the measured change in blood pressure between the two periods for the CO_2 sampling. For both measurement approaches the ± delta for CO_2 for hypotension are shown.

Figure 4A - CO_2 Delta ABG vs BP Delta

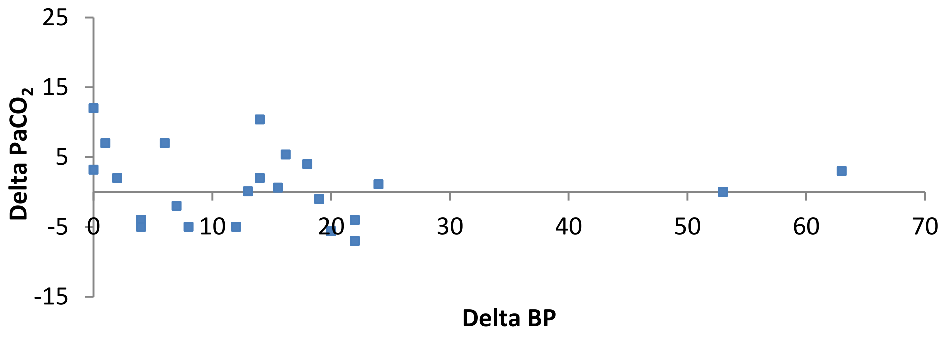

Figure 4B - CO_2 Delta ET vs BP Delta

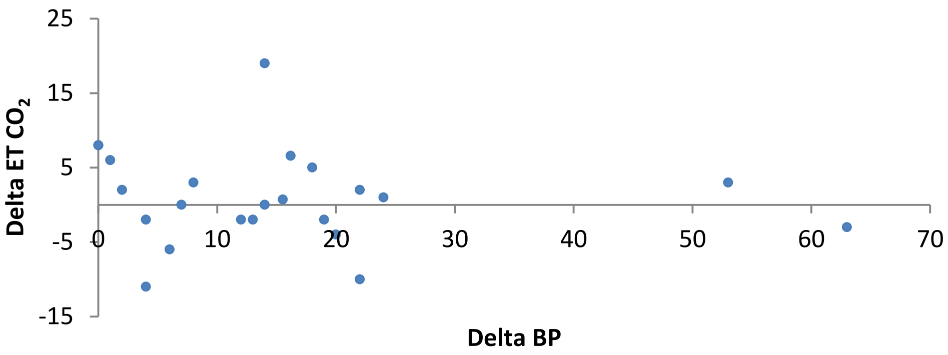

It is evident for the degree of hypotension that the basic trending with the two measures are visually similar.

To compare the response of the two measurement techniques with blood pressure controlled we have examined the correlation for these measures in Figure 5.

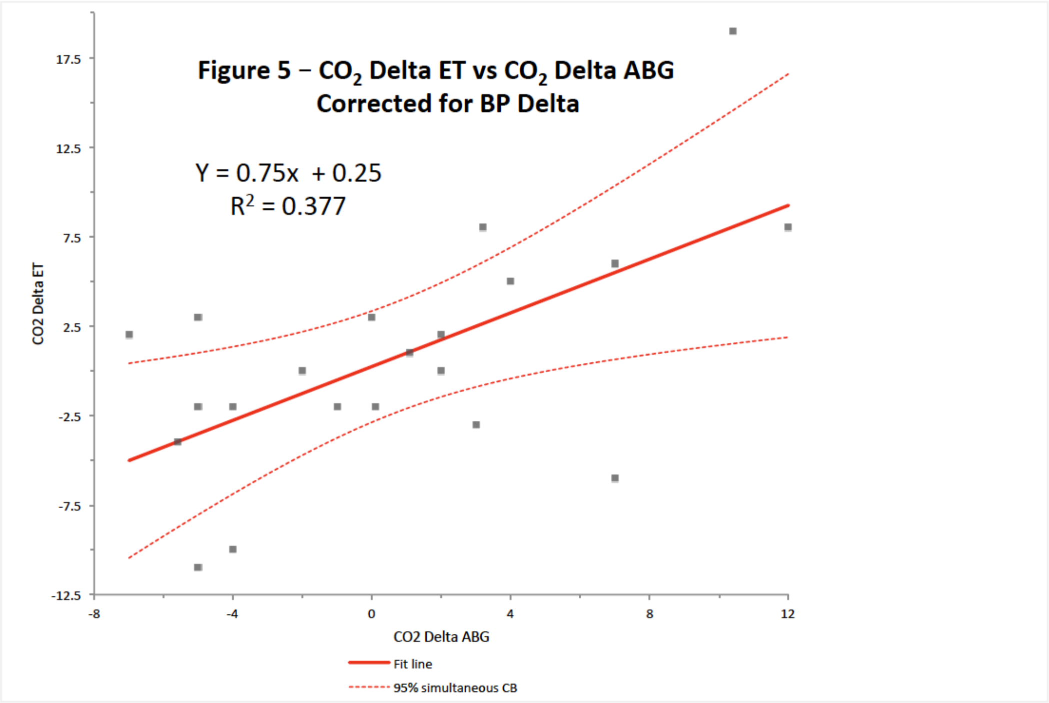

The correlation is now significantly better when BP is controlled compared to the data depicted in Figure 3. The correlation co-efficient is now 0.63 and the p-value 0.007. These data indicate that BP is an interactive term for the measurement of CO_2 intra-

operatively. Our cross-product results indicating an interaction between AUC for hypocapnia and hypotension would support this contention.

From the above analyses we would conclude that trending of $ETCO_2$ is a surrogate for $PaCO_2$ in this study. A prospective study whereby more rigorous data retrieval matching was undertaken for these measures would be a worthwhile pursuit. See manuscript text for further discussion of these issues.
