# Supplementary material for: End-Tidal Hypocapnia Under Anesthesia Predicts Postoperative Delirium
Source: Front Neurol. 2018 Aug 17;9:678. doi: 10.3389/fneur.2018.00678 (PMC6108130; doi:10.3389/fneur.2018.00678)
Supplement: Supplementary file 4 [file Data_Sheet_4.pdf]

Supplemental File 4:

Interplay between end-tidal CO_2 and blood pressure – An interaction between CO_2 and blood pressure has been demonstrate in this study as demonstrated from examination of Figure 3C. Not addressed in that figure is the potential dynamism of this interaction. The variability in end-tidal CO_2 and blood pressure at 1 hz is depicted below in a representative patient study.

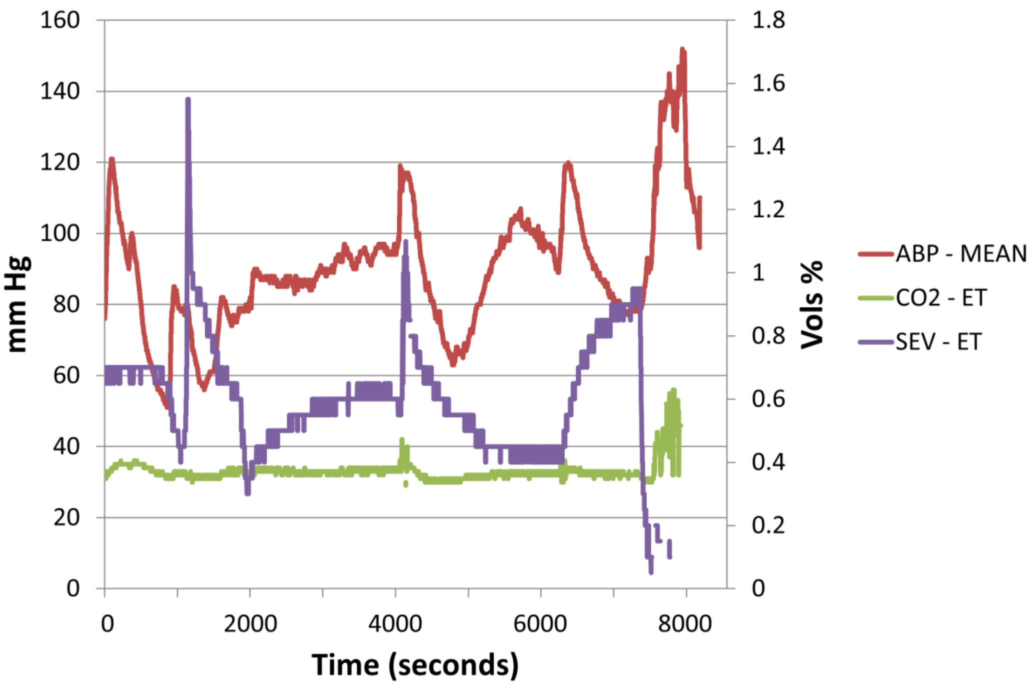

Periods of simultaneous hypocapnia and hypotension are seen in at least 3 time periods in this anesthetic course at about 900 seconds, 1200 seconds and 5000 seconds. Other periods of dynamism are seen at the end of the procedure where the opposite effect is seen – simultaneous hypertension and hypercapnia.

Recent work (Duffin et al., 2018) has highlighted the complexity of the relationship between alterations in end-tidal CO_2, CBF and resistance maps of this interaction. Very small changes in CO_2 can result in varied regional changes in CBF and vascular resistance (see figure below):

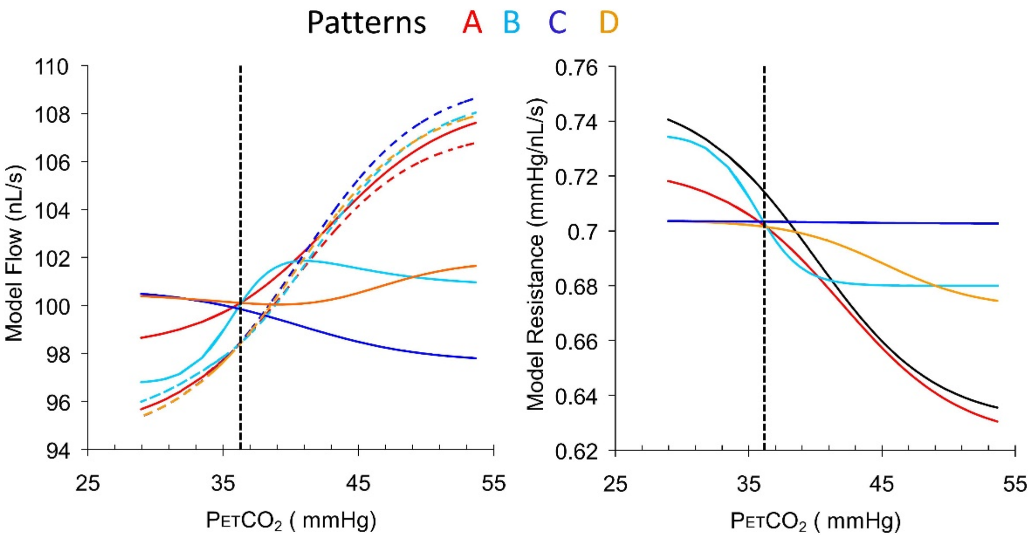

Four different response patterns have been identified in the figure above: Type A (colored red) – the normal increase in CBF with increasing CO_2 (BOLD response in the left figure as a surrogate for CBF and cerebrovascular resistance in the right figure with a sigmoidal response of increasing flow and decreasing vascular resistance with hypercapnia). Type B (colored cyan) has a normal initial response then is exhausted and becomes vasoparalyzed. Type C (colored dark blue) is the true representation of intracranial steal and is vasoparalyzed throughout the alteration in end-tidal CO_2 so resistance changes are passive. Type D (colored brown) is initially pressure passive and then demonstrates some CO_2 responsiveness with hypercapnia. The dotted vertical line is the patient's resting end-tidal CO_2 so it can be seen that small intraoperative changes in CO_2 from the patient's baseline status post induction of anesthesia can result in varied regional alterations in CBF and cerebrovascular resistance. Small changes in CO_2 are easily evident during conduct of anesthesia with a surgical procedure as demonstrate in this study. Alterations in blood pressure would further complicate these interactions. Importantly these changes can occur on a microregional basis contributing to a very dynamic situation during the course of an anesthetic.

Reference:

Duffin, J., Sobczyk, O., McKetton, L., Crawley, A., Poublanc, J., Venkatraghavan, L., et al. (2018). Cerebrovascular resistance: the basis of cerebrovascular reactivity. *Front. Neurosci.* 12, 409. doi:10.3389/FNINS.2018.00409.
